# Understanding the Role of Psychosocial Factors in Pakistani Parents’ Hesitancy to Vaccinate Their Kids: The Mediating Role of Knowledge and Mistrust of Science about the COVID-19 Vaccine

**DOI:** 10.3390/vaccines10081260

**Published:** 2022-08-05

**Authors:** Riffat Shahani, Jianxun Chu, Olayemi Hafeez Rufai, Asma Zawar, Sayibu Muhideen, Sana Dilawar, Tunde Simeon Amosun

**Affiliations:** 1 School of Humaities and Social Sciences, University of Science and Technology of China, Hefei 230052, China; 2 School of Public Affairs, University of Science and Technology of China, Hefei 230052, China

**Keywords:** vaccine hesitancy, parents’ intention, vaccine knowledge, mistrust in science, conspiracy belief

## Abstract

Vaccination is a vital component in the battle against outbreaks of infectious diseases. Recognizing parents’ reluctance to vaccinate their children is even more critical now, given the ongoing threat of a COVID-19 pandemic. Conspiracy theories, vaccination safety concerns, parental efficacy and risk perception, and a lack of confidence in science all influence intention. To investigate how these variables interact with vaccination behavior against COVID-19, we developed a model with psychosocial factors serving as the predictor and mistrust in science and vaccine knowledge serving as the mediator. In order to validate the model, the parents’ intentions regarding their children’s vaccination with COVID-19 were used. The study included 454 Pakistani parents who completed an online questionnaire assessing their intention to vaccinate their children. We analyzed the data using structural equation modeling (SEM). A significant level of vaccine hesitation is due to belief in vaccine conspiracy theories, and vaccine safety concerns were investigated. A surprising correlation exists between risk perception and vaccination intentions, followed by parental self-efficacy. It is significant to note that vaccine knowledge mediated conspiracy beliefs, risk perceptions, and intentions fully but partially mediated parental self-efficacy. Conspiracy theories were mediated by a mistrust in science, while perceptions of risk and vaccine safety were partially mediated. The findings of this research were used to develop sensible policy reforms and public health campaigns to encourage vaccination against both common infections such as measles, human papillomaviruses, or pertussis, and novel diseases such as COVID-19.

## 1. Introduction

One of the most important achievements in modern medicine is the development of vaccines, which contribute to saving lives by preventing the spread of infectious diseases. To achieve herd immunity and avoid an outbreak, vaccinations must be administered to 80–90 percent of community people [1]. Extensive clinical and epidemiological research has demonstrated the positive benefits of immunization, such as illness elimination and reducing outbreaks and death rates. Additionally, vaccination reduces disease’s economic and social impact, improves the overall quality of life, and promotes gender equality [2]. Most parents worry about vaccine safety and efficacy [3,4]. Vaccine hesitancy and refusal have led to decreased vaccination coverage [5,6] and increasing epidemics of infectious diseases [7,8]. Many parents and caregivers are no longer aware of formerly feared contagious illnesses that have been effectively eradicated through vaccination [3,9]. Recent research examined the health belief model using a representative sample from the United States of America. According to this poll, 49.45 percent of parents want the COVID vaccination for their Kid, and 44.17% intend to vaccinate against COVID-19 whenever it becomes available. Concerns about adverse vaccination effects (61.5%) and vaccine safety (48.96%) were key factors contributing to vaccine hesitation [10]. According to another study, 1 in 5 parents is vaccine-hesitant about the COVID-19 vaccine [11].

Vaccine safety is a severe concern for parents’ intention. It is a barrier to the parental choice in taking the vaccine. Vaccination is recommended by most health professionals around the globe [12,13]. Healthcare providers are the most frequently used and trusted source of information regarding vaccines for parents, including those who are vaccine hesitant. However, parents are increasingly concerned that vaccines might harm their children, particularly when it comes to vaccine safety [14]. Some vaccine safety concerns have been limited to specific regions. For example, the Hepatitis B vaccine suspended in France in the 1990s to a purported link between the vaccine and multiple sclerosis [15]. There have also been other global concerns, such as the purported (and now thoroughly discredited) link between the measles, mumps, rubella vaccine, and autism [16]. In other countries, concerns about vaccine safety have led to declines in vaccination rates and an increase in disease incidence [17]. The results of this study examine parents’ beliefs about vaccine safety and their intention to vaccinate against COVID-19.

Self-efficacy refers to a person’s belief in their ability or effectiveness in a particular field [18]. In addition, self-efficacy is also a person’s belief that he can control and decide on specific actions that will lead to specific results [19]. So, it can be said that self-efficacy is the level of a person’s confidence that he can do something to achieve a goal. Parents with a strong sense of self-efficacy are expected to vaccinate their kids without hesitation. Research indicates that self-efficacy is favorably associated with vaccination uptake [20]. Understanding that health practices such as vaccination can avert adverse outcomes may not be sufficient to encourage behavioral change [21,22]. Health belief theories (HBTs) claim that feeling competent in carrying out such activities is vital for behavioral change [23]. In theory of planned behavior (TPB), this perceived competence is perceived behavioral control, whereas, in extended parallel processing model (EPPM), it is referred to as self-efficacy [24]. Due to these commonalities, recent TPB research often operationalizes perceived behavioral control as self-efficacy [25].

Research indicates that risk perception is a subjective psychological construct affected by cognitive, emotional, social, cultural, and individual variation across persons and between countries in the wake of a public health crisis [26]. Regardless of how it is defined and varied, substantial scientific evidence shows that correct general risk perceptions are crucial for controlling public health concerns efficiently. The same is true for relationships between COVID-19 risk perception and reported protective behaviors, such as the greater the degree of risk perceived, the more persons report practicing protective behaviors [27]. While it is critical to assess the danger of a public health catastrophe appropriately, overestimation may result in unintended effects such as worry and stress. According to a systemic review conducted by Schmid, Rauber [28] on barriers of influenzas vaccination, 42 studies indicated that a low risk perception was a critical factor affecting vaccine intention. Some studies also show that parents who have a low-risk perception of their children being ill from vaccine-preventable diseases are more likely to refuse vaccination [29,30,31].

A previous study indicates that worry and stress caused by risk perceptions are significant elements of the probability of believing in conspiracy theories [32]. Due to people’s skepticism and conspiracy theories, vaccination rates in developing countries like Pakistan are low. For instance, Pakistan have a long history of believing in health-related conspiracy theories. Since the beginning of COVID-19, conspiracy belief in Pakistan is rampant which influences people’s health centric behavior towards vaccination [33,34]. As [35] found out that COVID-19 vaccination experiences a sporadic increase in its hesitancy rate due to misinformation. For example, misinformation related to the COVID-19 vaccine containing nanochips controlled via the 5G frequency spectrum and implanted in human bodies [36]. Thus, the continued presence of the COVID-19 is influenced by conspiracy beliefs which have immediate consequences on vaccine hesitancy [37].

Building trust is essential in combating the current COVID-19 epidemic. In addition, it is associated with a higher level of compliance with health recommendations such as social distance and lockdown measures [23]. Extant literatures have shown that mistrust in science is a significant element in the decision-making process relating to vaccination. Most studies [38] have examined trust in vaccinations, but mistrust of science has received less attention.

Indeed, increased health knowledge is connected with more favorable views regarding vaccination in general [39]. Previous study shows that adequate knowledge of COVID-19 is associated with a favorable attitude toward preventative actions [40]. Lack of scientific knowledge might exacerbate public mistrust in science [41]. Nonetheless, there is inconsistent findings about knowledge and intention to vaccination [42]. Educational campaigns may be an excellent way to fill in knowledge gaps and correct misinformation about COVID-19 vaccines. Such campaigns may define a vaccination as a formulation containing a synthetic, dead, or weakened version of the disease-causing agent [43].

**Figure 1:**
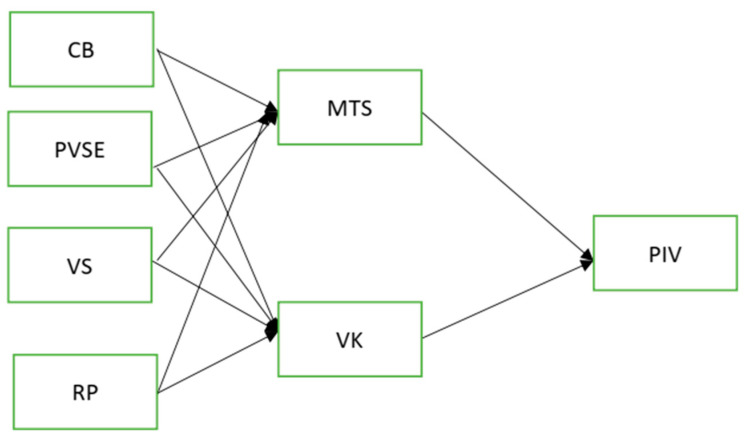
Research framework. Conspiracy belief (CB), Vaccine Knowledge (VK), Vaccine Safety (VS), Risk Perception (RP), Parental Vaccine Intention (PIV), Parental Vaccine Self-Efficacy (PVSE) and Mistrust in Science (MTS).

According to the above discussion, the present study aims to explore the psychosocial factors that influence parents’ intention to vaccinate their kids. In addition, it will investigate the elements affecting the ‘vaccine hesitancy’ phenomenon. The majority of studies have examined the effect of specific psychosocial variables on vaccination intention, primarily in the general population e.g., [44,45]. Such studies are beneficial to aid in understanding the choice to vaccinate kids. However, they do not provide a comprehensive analysis in investigating the psychosocial factors influencing their decision, especially in the case of persons who self-identify as ‘anti-vaxxers’ parents. Such groups may differ in their intention to prevent vaccination, which may have significant consequences for understanding vaccination behavior. For this purpose, we developed a model that identifies the following fundamental factors: conspiracy belief, vaccine safety concern, parental self-efficacy, and risk perception, which could positively lead from vaccine knowledge to parents’ intention, and negatively lead from mistrust in science of parents’ intention to vaccinate their kids. To the best of the authors knowledge, no prior research has been conducted on psychosocial factors that could impact parents’ intention to vaccinate their kids in Pakistan. Our hypothesis is summarized in Figure 1. 

## 2. Material and Methods

### 2.1. Participants

To validate the suggested conceptual framework and test the study hypotheses, a questionnaire was prepared and distributed through the Internet to collect data from residents of Pakistan. Four hundred and fifty-four Pakistani parents participated in a survey of vaccination behavior. The sole requirement for inclusion was that at least one kid is within the vaccine age range (0–10 years). After being told about the research’s anonymity and completing the informed permission forms, a convenience sample of 454 parents (31.7 percent females, 68.3 percent males) completed an online self-report questionnaire explicitly designed for this study. The survey was distributed through social media sites, including WhatsApp, Facebook groups, Twitter, and Instagram. Before uploading the questionnaire link, the group administrators were informed of the study’s goal and granted permission to share the information. All questions have been updated using a five-point Likert scale ranging from strongly disagree (1) to strongly agree (5). Pilot research with 30 participants was conducted to ascertain that the questions and response formats were comprehended. The majority of respondents said that the language used was understandable and that the questionnaire was also appropriate in length.

### 2.2. Measures

Demographic data and prior behavior: Participants were questioned about their age, gender, number of children, religion, and city of residence. Additionally, parents were requested to disclose their prior vaccination-related behavior (whether their children received mandatory vaccinations, booster shots of compulsory vaccinations, influenza vaccinations, and non-mandatory vaccinations).

### 2.3. Predicting and Predictor Variables

#### 2.3.1. Parents’ Intention towrds COVID-19 Vaccine

Intention of parents towards COVID-19 vaccinaion for kids was assessed through 4 items using a Likert scale ranging from *‘Strongly disagree’ (1) to ‘Strongly agree’ (5) (e.g., ‘I intend to vaccinate my son/daughter’*); adapted from [46].

#### 2.3.2. Mistrust in Science about COVID-19

Mistrust in science about COVID-19 vaccination was assessed with the 5-item belief on the COVID-19 scale [47]. The scale was developed to measure mistrust of science. Participants were asked to indicate their degree of agreement with the items on a Likert scale from strongly disagree (1) to strongly agree (5). A sample item is “*I don’t trust the information about the virus from scientific experts*.”

#### 2.3.3. Vaccine Knowledge

COVID-19 Vaccine knowledge scale adopted from [48]. Vaccine knowledge questions were designed based on practical vaccine-related information relating to the public health benefits of vaccination. The six questions were scored on a 5-point Likert scale (strongly agree, agree, neutral, disagree, strongly disagree).

#### 2.3.4. Risk Perception

Risk perception was evaluated using the COVID-19 Perceived Risk Scale [49]. The scale includes four items for the cognitive dimension of perceived risk (e.g., “There is a high likelihood of acquiring COVID-19 in general”) and four items for the affective dimension (e.g., “*I am worried that I will contract COVID-19”).* All things were assessed on a scale from (1) Strongly disagree to (5) Strongly agree.

#### 2.3.5. Self-Efficacy

Based on previous research [50], four items on a 5-point scale (1 = strongly disagree, 5 = strongly agree) were used to assess respondents’ perceived self-efficacy in getting vaccines for their kids (e.g., it would be easy for me to get effective vaccines for my children).

#### 2.3.6. COVID-19 Vaccine Safety

COVID-19 vaccine safety assesed on four items using 5 point likert scale (e.g., “I worry about the short term side effects of the COVID-19 vaccine”; adapted from [51] on a Likert scale ranging from (1) disagree entirely to (5) completely agree.

#### 2.3.7. Conspiracy Beliefs

COVID-19 vaccine conspiracy beleifs were assessed by adapting the 10-item Anti-vaccine conspiracy belief scale [52]. Participants were asked to indicate their degree of agreement with the items on a Likert scale from strongly disagree (1) to strongly agree (5). A sample item is *“Immunizations allow governments to track and control people.”*

### 2.4. Statistical Analysis

Statistical analysis was performed using two different statistical packages, SPSS 26.0 and AMOS 24. The data pre-processing eliminated 46 responses due to missing data points. As a result, this research analyzed just 454 individuals’ responses. First, a demographic analysis of the respondents was performed to better understand the sample structure. Second, the measurement model is used to assess the relationship between latent variables and their measures. To examine the measurement model, exploratory and confirmatory factor analyses were conducted. Finally, structural equation modeling provides insight into the relationship between predictors and dependent variables. Path analysis can determine the effects of vaccine knowledge and mistrust of science regarding COVID-19. 

## 3. Result

### 3.1. Descriptive Statistics

A total of 454 Pakistani parents participated in the survey; most of the respondents were females (68%). The majority of respondents (54.8%) possess a master’s degree and are highly educated. Approximately 64 percent of parents have only one or two children. Approximately 23.1% of the children were under one year of age, 31.7% were 1–3 years of age, 19.8% were 4–6 years old, and 12.1% were aged 7–9 years. A large majority of respondents were middle class, with an annual income of 1000–10,000 dollars. There is a wide range of professions represented by parents, with engineers (33.9% of all parents) making up the highest percentage. More than seventy percent of the population lived in urban areas. Among the parents, 64.2% had prior experience administering vaccines to their children, while 35.2% had no prior experience. Detailed descriptive statistics, such as percentage and frequency are presented in Table 1.

The next step after descriptive statistics was to analyze the internal consistency of the data. In order to accomplish this, we perform exploratory factor analysis. The EFA is analyzed using Cronbach’s alpha (CA), factor loading, composite reliability (CR), and average variance extracted (AVE). Table 2 summarizes Cronbach’s alpha (CA) and factor loading whereas Table 3 presents CR and AVE. Cronbach’s alpha was used to test the internal consistency of the survey items. Factor loadings indicate the variance explained by a variable on a particular factor. The cutoff points for Cronbach’s alpha > 0.7, factor loading > 0.5, composite reliability > 0.7, and AVE > 0.5.

### 3.2. Measurement Model

#### 3.2.1. Exploratory Factor Analysis (EFA)

To establish the measurement model, we conducted an exploratory factor analysis using SPSS, followed by Kaiser-Mayer-Olkin (KMO) measures and Bartlett’s test of sphericity to ensure our measurements were adequate. Bsed on the value of KMO = 0.92 as well as Bartlett’s sphericity test (χ^2^ = 1080.65; df = 391; *p* < 0.01). An exploratory factor analysis was performed based on promax rotation. In order to determine the factor loadings and validity of all items using the principal component technique and varimax rotation, a suppressed value of 0.50 was used to calculate all loadings. In all cases, the loadings exceeded the minimum threshold of 0.50 and no cross-loading effects were observed. As a result of the research and the data collected, it can be concluded that the scales and dimensions employed in the study are appropriate. These dimensions are suitable for the study. Table 2 presents the factor loadings and Cronbach’s alpha result. To provide a robust investigation, the factor loadings were greater than 0.6, as shown in Table 2. 

According to [53] in this study, the Cronbach alpha values were all greater than 0.70. To determine the internal consistency of constructs, Table 3 shows composite reliability (CR) and average variance extracted (AVE). CR values greater than 0.70 indicate that the conceptual framework has a reasonable degree of internal consistency. Furthermore, all constructs had AVE values exceeding 0.50. Additionally, all constructs had higher CR values than AVE, explaining the variance in the average scores, and the composite reliability indicated that all constructs are valid (see Table 3). Further, discriminant validity is a critical criterion for determining credibility. To be considered valid, the AVE of a construct must be greater than the squared correlation between that construct and the other constructs. According to Table 3, the measurement model indicates that discriminant validity is satisfactory and affirmed by Table 4 correlation matrix. Diagonal elements represent multiple squared correlations between variables. Table 3 indicates that AVE values range from 0.615 to 0.808. Additionally, the diagonal value ranges from 0.696 to 0.899, which indicates that the diagonal variable is greater than the other AVE values, indicating excellent discriminant validity for all constructs. The measurement model revealed that discrimenent validity is satisfied and affired Table 4 correlation matrix. Table 4 presented that the intercorelations for all the variables were statistically significant, which indicated that structural equation modeling approach was appropriately conducted to examine the conceptual framework. An overview of correlation can be found in Table 4 below.

#### 3.2.2. Confirmatory Factor Analysis (CFA)

This measurement model (CFA) was evaluated to determine whether it had a sufficient level of fitness. For the confirmatory factor analysis, we used AMOS 24, which employed a maximum likelihood approach to uncover relationships among latent variables. Absolute fit indices such as “ χ^2^ /df (Chi-square to degree of freedom ratio) and RMSEA (root mean square error of approximation) were used; incremental fit indices such as CFI (comparative fit index) and TLI (Tucker–Lewis Index) were used; and parsimony fit was evaluated using AGFI (adjusted goodness-of-fit index)”. The model fit is good when “χ^2^/df < 3.0, with the values of CFI, TLI > 0.90, RMSEA < 0.08, PClose > 0.05; SRMR < 0.08 and AGFI > 0.80” [30]. The fit statistics for the measurement model obtained were “(CMIN = 997.032,df = 381, p.000; X^2^/DF= 2.617; CFI = 0.947; TLI = 0.935; RMSEA = 0.058)” and were all more than the minimum acceptable limit [54,55].

After validating and confirming the measurement model, the second phase involved estimating the structural model that captured the predicted relationships between exogenous and endogenous variables. In order to determine the goodness of fit (GOF) of the structural model, we used the same criteria as for the measurement model. The overall fit of the structural equations was evaluated using the following criteria: *p*-value, CHI/DF, NFI, IFI, RFI, TLI, CFI, GFI, AGFI, and RMSEA [56]. To establish the significance of the indirect effects, standardized direct and indirect path coefficients were examined using bootstrap analysis utilizing a 2000 bootstrap sample and a 90% confidence interval (CI) [57].

### 3.3. Structural Equation Modeling

**Figure 2:**
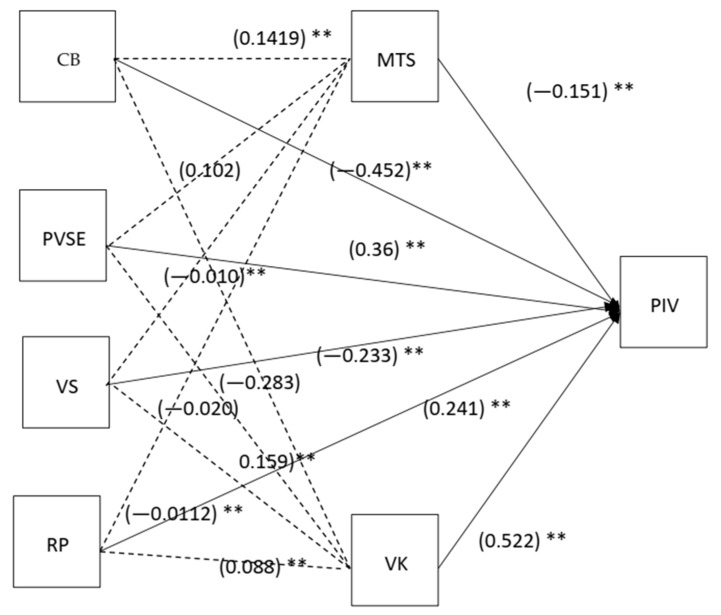
Diagram of structural model with standardized regression coefficients. Note: Straight line (**→**) represent direct relation, Dotted line (----) represent mediation. Significance of Estimates. *** *p* < 0.001. ** *p* < 0.010. * *p* < 0.050. *Conspiracy belief (CB), Vaccine Knowledge (VK), Vaccine Safety (VS), Risk Perception (RP), Parental Vaccine Intention (PIV), Parental Vaccine Self- Efficacy (PVSE) and Mistrust in Science (MTS).

In order to examine the main research hypotheses among the proposed constructs, structural equation modeling was employed. To examine the fitness of the structural model, all fit indices were determined to be within acceptable limits: TLI = 0.929; IFI = 0.973; GFI = 0.982; AGFI = 0.947; NFI = 0.951; CFI = 0.972; and RMSEA = 0.050, PClose = 0.478; RMR= 0.0382 [54,56]. These indices illustrate a well-fitting model that accounts for 55% of the heterogeneity in participants’ intentions to receive the COVID-19 vaccine. Based on path coefficient analyses, we were able to determine the main causal pathways, as shown in Table 5. This path analysis is illustrated in Figure 2 to demonstrate the significance and relevance of each hypothesis. According to the proposed conceptual framework, hypothesis 1 examines the effects of psychosocial variables on the intention of parents to vaccinate their children. Based on the results of the study, conspiracy beliefs (H1a) and mistrust of science about COVID-19 (H1e) have negative and significant effects on parents’ intentions, (β_CB__→PIV_ = −0.452, t = 2.967, *p*< 0.010; β_MTS__→PIV_ = −0.151, t = 4.135, *p* < 0.001) supporting H1a and H1e. In contrast, the direct impact of risk perception (H1d) and vaccine knowledge (H1f) on vaccine intention is positive and significant (β _VK__→PIV_ = 0.52, t = 14.343 *p* < 0.001; β _RP__→PIV_ = 0.241, t = 4.135, *p* < 0.005), supporting H1d and H1f. However, there was no significant effect of parental vaccine safety concerns about COVID−19 vaccine (H1b) and parental self-efficacy (H1c) on COVID-19 vaccine intention, (β _VS__→PIV_ = −0.233, t = 2.889, *p* > 0.05; β _PVSE__→PIV_ = 0.368, t = 1.969, *p* > 0.090), not supporting H1b and H1c.

#### Mediation Test

In order to test the mediating effect of vaccine knowledge and mistrust in science about COVID-19, the bootstrapping method was used. Table 6 illustrates the direct and indirect effects of vaccine knowledge and mistrust in science on parents’ intention to vaccinate their children. The table also includes a column for full, partial, and no mediation. As a result of the presence of a mediator, the direct effect of predictors on the outcome variable becomes insignificant [58]. Partial mediation implies that some effects of predictors pass through mediator variables while other effects pass directly from independent variables to dependent variables and have *p*-values of 0.005 [59]. We investigated that vaccine knowledge of parents and their mistrust in science about COVID-19 mediates between conspiracy belief, parental self-efficacy, vaccine safety concerns and risk perception and parental intention towards their kid’s COVID-19 vaccination. Hypothesis 2 tested the mediating effect of vaccine knowledge between the psychosocial variable and intention towards their kid’s vaccination. The indirect effect of vaccine knowledge between conspiracy belief (H2a), parent vaccine self efficacy (H2b) and risk perception (H2d) on parent vaccine intention was significant (β _CB_ _→VK__→PIV_ = −0.28, *p* < 0.01; CI [−0.127, −0.06]; β_RP__→VK__→PIV_ = 0.088, *p* < 0.01; CI [0.042,0.139]; β _PVSE__→VK__→PIV_ = 0.157, *p* < 0.01; CI [0.099,0.234]). Vaccine knowledge negatively mediated H2a and positively mediated H2b and H2d. While, vaccine knowledge was not significantly mediting between vaccine safety concern and parent vaccine intention (H2c) (β _VS__→VK__→PIV_ = −0.020, *p* > 0.05; CI [−0.063,0.019]), H2c not supported. Hypothesis 3 examined the mediating impact of mistrust in science between psychosocial factors and vaccine intention of parents. The indirect impact of misturst in science between conspiracy belief (H3a), vaccine safety concerns (H3c) and risk perception (H3d) and parents’ intention to vaccinate their kids were significant (β CB→MTS→PIV = 0.1419, *p* < 0.05; CI [0.02,0.263]; β_VS__→MTS__→PIV_ = −0.010, *p* < 0.05; CI [−0.341, 0.031; β_RP__→MTS__→PIV_ = −0.0112, *p* < 0.05; CI [−0.36, 0.062]) H3a, H3 c and H3d supported. PVSE and PIV (β_PVSE__→MTS__→PIV_ = 0.102, *p* >0.05; CI [−0.245, 0.356]) was not significantly mediated by MTS therefore H3b rejected.

## 4. Discussion

Vaccination is an important component of public health, particularly in children, as it is a primary prevention strategy for infectious diseases. COVID-19 is highly contagious with a complex transmission pattern. Children must be vaccinated in order to protect themselves against COVID-19 and other diseases common in the general population. Parents are responsible for determining the level of vaccination coverage for their children. Thus, monitoring vaccination awareness and understanding among parents, who are the primary target group for COVID-19 immunization campaigns. In this study, psychosocial factors were investigated in relation to parental intention. As a mediator between psychosocial factors and intention to receive vaccination, vaccine knowledge and mistrust of science about COVID-19 were examined. To the extent of our knowledge, this is one of the few studies designed to collect extensive data on parents’ awareness, mistrust in science, and intention to get COVID-19 vaccines.

In the current study, we aimed to determine parents’ intentions to vaccinate their children under 10 and identify psychosocial predictors of those intentions. The intention of parents to vaccinate their children has been the subject of few studies so far. Most of these studies were conducted prior to the release of the COVID-19 vaccine for children between the ages of 12 and 18. According to the current study, nearly one-third of parents (39.30 percent) have expressed an intention to vaccinate their children against COVID-19 if the vaccine becomes available. It is consistent with the findings of a recent study (Galanis et al., 2020). We uncovered alarming statistics regarding parents’ intentions who participated in our survey. According to the survey, half of the respondents said they would not vaccinate their children when the vaccine became available. In terms of psychological factors, descriptive statistics revealed that parents expressed low to moderate levels of vaccination intention. Parental self-efficacy and vaccination knowledge were positively associated with vaccine intention. Whereas vaccination intention is inversely correlated with conspiracy beliefs, risk perception, mistrust in science, and vaccine safety concerns. In the present study, it was demonstrated that conspiracy belief and parental intention towards vaccination (H1a) were significant and supported. Distorted views about vaccination are strongly associated with misinformation about the risks posed by vaccination, leading parents to believe the conspiracies. In order to increase the acceptance of vaccines, it is essential to provide parents with adequate information about the risks and safety of the vaccine, as well as solid evidence regarding its efficacy.

According to the results of this study, risk perception and parental intention are positively correlated (H1d). There has been a positive and significant relationship between risk perception and preventive health behaviors [60], particularly during pandemics [61,62,63]. Based on previous research and theories, it is not surprising that risk perception influences behavioral intentions. Additionally, a study by Thompson [64] demonstrated a positive relationship between COVID-19 risk perception and intention. As a result of our study, we have gained new insight into why risk perception plays an important role in the promotion of preventive behavior. The study demonstrated that public risk perceptions make parents more conscious of adopting health-conscious behaviors since high-risk perceptions lead to preventive actions in many infectious disease outbreaks and have improved epidemic control [65]. Thus, the result of the current study was significant (H1d).

In contrast, parental self-efficacy and vaccination safety concerns are not significantly associated with intention to vaccinate against COVID-19 (H1b and H1c). The absence of a statistically significant association may be due to the fact that immunizations were not yet widely available to the general public. The transtheoretical model (TTM) of behavioral changes suggests that differences in self-efficacy occur at a later stage of health decision-making. We may not be able to establish a link between self-efficacy and vaccination intention due to the low variation in self-efficacy [66]. At that stage, your primary concern is regarding vaccine safety when you are unable to make a decision about whether to get the vaccine or not. As a result of this study, it has been concluded that vaccine safety for COVID-19 vaccine (H1d) has not been established. [67]. The results of a Canadian focus group study [68] also indicated that participants were reluctant to accept vaccines during future pandemics due to perceived uncertainties regarding the safety and seriousness of new vaccines. Despite the availability of more information on the safety and effectiveness of vaccinations, the proliferation of misinformation on social media is a serious concern. The refusal to receive the vaccination was based on a specific set of beliefs that reflected a broader distrust of vaccines [69].

Based on the findings of this study, parental knowledge (H1f) is positively associated with parental intention to vaccinate their children, whereas parental mistrust of science about COVID-19 (H1e) negatively correlates with parental intention to vaccinate. This study supported H1e and H1f. In our research, we found that parents who are well informed about vaccines are more likely to vaccinate their children when these vaccines become available [70]. COVID-19 is contagious and exhibits a complicated transmission pattern. The lack of a COVID-19 vaccine in Pakistan particularly for children under the age of 12 makes them more susceptible to the virus, facilitating the spread of the epidemic. Children should be vaccinated not only to protect themselves against COVID-19 but also to protect other vulnerable groups. Based on our findings, it may be concluded that knowledge is a prerequisite for self-efficacy and intention. In order to improve these constructions, we must first raise public awareness about the health issue. According to previous research, a high level of vaccination knowledge significantly influences vaccination intentions and uptake [39]. In many cases, vaccination reduces the transmission of pathogens (viruses, bacteria) and increases their likelihood of eradication [71]. Prior to the invention of vaccinations, children were constantly at risk of death and disability from diseases such as measles, polio, tuberculosis, diphtheria, and rubella. We can learn from history that if we want to prevent pandemics such as COVID-19, we must educate people about infectious diseases and how vaccination programs can help control outbreaks [42].

In our study, we found that parents’ mistrust of science affected their intention to vaccinate their children. It is possible that parents may be hesitant to vaccinate their children due to inaccurate information circulating regarding the COVID-19 vaccination. Sunstein and Vermeule [72] stated that mistrust in science is a significant predictor of vaccination hesitation among parents in Canada, the United States, and the United Kingdom. Similarly, parents with a lack of confidence in their primary care provider are more likely to seek vaccine information online, resulting in vaccine rejection [73]. It’s easier to spread conspiracies online than it is to provide accurate information. In general, people who are misguided against vaccination do not believe in scientific findings. A recent study conducted in the United States indicated that mistrust is a prevalent factor underpinning plans to avoid the COVID-19 vaccination program, with 55 percent of respondents stating that they lack trust in science to ensure vaccine safety and efficacy [42].

### 4.1. Mediating Effects of Vaccine Knowledge

In this study, the findings indicate that anti-vaccine conspiracy theories are fully mediated (H2a) by vaccine knowledge concerning vaccination intentions for parents. Conspiracy theories are more likely to influence individuals’ views about vaccines to the detriment of scientific consensus. Thus, our findings suggest that conspiracy beliefs are hindering the development of trust in health problems and the acceptance of scientific knowledge. Conspiracy beliefs and parental intention to vaccinate their children were completely mediated by vaccine knowledge. It is shown that a full mediation occurs when the direct effect of conspiracy belief becomes insignificant as vaccine knowledge is introduced as a mediator. In fact, conspiracy theories undermine knowledge of vaccinations, a consequence that many people are unaware of until they are faced with it. The consequences of these beliefs are even more serious for public health since parents who use scientific knowledge to improve their behavior are more likely to be successful. It has been shown that parents who understand the scientific knowledge about COVID-19 vaccines are more likely to vaccinate their children and are less inclined to believe anti-vaccine conspiracy theories [47].

Parents who cared more about their child’s health rated COVID-19 risk as greater, checked statistics more frequently, and demonstrated a stronger behavioral intention to engage in preventative activities. As a result of our research, it has been determined that risk perception (H2d) is fully mediated by vaccine knowledge. It was common for parents to seek vaccination information in order to prevent infection. It was perceived by parents that obtaining adequate vaccination knowledge was a high-risk activity. Ultimately, these parents are more likely to adopt COVID-19 vaccinations for their kids. Since the idea of ‘risk’ is socially and culturally constructed Askelson, Campo [46] demonstrate that parents with inadequate knowledge may underestimate the dangers of certain illnesses in their children. Therefore, it is imperative that parents become more knowledgeable about COVID-19 vaccines and become more educated about vaccinations. In addition to protecting their children, parents also strive to prevent the spread of infectious diseases within their communities. They also indirectly safeguard those who have not been vaccinated or are not able to be vaccinated. The relationship between parental self-efficacy (H2b) and vaccination intention was partially mediated by vaccine knowledge [74]. Observations from our mediation study indicate that parents’ intentions were more closely related to how they felt about their knowledge (e.g., feeling appropriately educated about vaccination and understanding of the value of immunization) than to what they knew objectively (knowledge of the COVID-19 vaccine and vaccine-preventable diseases). It is possible that these promising results reflect a feeling of considerable parental self-efficacy rather than expertise.

In this study, it has been demonstrated that vaccination safety concerns (H2c) are related to lower COVID-19 vaccination coverage in children. It is essential to have knowledge about vaccine safety, but this alone is not sufficient to change people’s perceptions of vaccine safety [75]. Especially when the different varients of COVID-19 affect the vaccine’s effectivness. This reduces the trust of parents on vaccine safety. Therefore, vaccine knowledge has not mediated the relationship between vaccine safety and parent intent. A number of studies have demonstrated that people who are already skeptical of vaccination may be adversely affected by explicit education and advocacy of vaccination. There is a possibility that excessive information may cause hesitancy. Whenever people come across information that contradicts their values, they may experience fear and respond defensively, resulting in resistance, reinforcing their previous views, and decreasing their likelihood of participating in the desired activity (such as accepting vaccines) [76]. To facilitate vaccination acceptability, we must develop communication strategies that minimize the unexpected negative consequences of well-intentioned interventions. The goal of addressing vaccine hesitancy through communication requires a combination of proven, evidence-based, and context-sensitive approaches.

### 4.2. Mediating Role of Mistrust in Science

This study investigates the mediating role of mistrust about COVID-19 between psychosocial factors and the intention to vaccinate children. The mistrust of science fully mediated the relationship between conspiracy beliefs and COVID-19 vaccination intentions of parents (H3a). Science mistrust increases the influence of conspiracy theories and decreases the willingness of parents to vaccinate their children. On the other hand, mistrust on science might be a foundation upon which belief in conspiracy theories can flourish, fueled by existential dangers [77]. It is true that nervous parents may have an exaggerated reaction to hazards because of their psychological need to feel safe [78]. Apparently, this anti-vaccine conspiracy theory introduces a significant amount of misinformation, resulting in decreased vaccination rates and a negative effect on public health. Additionally, confidence in conspiracy theories, including scientific misinformation, may undermine trust in official medical sources and providers.

The results of our study indicate that mistrust of science has no mediation effect on parents’ self-efficacy and intention to vaccinate their kids against COVID-19. This interesting finding was revealed by our study. According to the results of this study, parents who are concerned about the efficacy of the vaccine (H3b) are less likely to have a positive intention toward the vaccine. Vaccination intentions were less likely to be positive in parents who delayed vaccination until a large number of other parents had received the vaccination and they had observed that the vaccine did not cause any harm to the vaccinated kids. It is consistent with the findings of other international contexts [74,79]. Additionally, this study revealed that mistrust of science partially contributes to vaccine safety (H3c). The findings of this study indicate that there is an increase in concerns regarding vaccine safety due to mistrust of science. Misinformation concerning scientific facts affects people’s perception when they are exposed to it. Ultimately, this leads to more concerns about the safety of vaccines. The results of our study are in accordance with the previous literature. According to other research, a diminished belief in science contributed to increased fear regarding the safety of the H1N1 vaccine in the United State [80,81]. When new vaccines come out on the market, safety concerns of people increase. There was increased concern and worry expressed by participants in three studies regarding the safety, efficacy, and long-term consequences of the newer vaccines [82,83]. A Canadian study suggests that authorities and media sources frequently misrepresent the risks associated with vaccination [84]. As a result, individuals developed a negative attitude toward vaccinations, resulting in a decrease in vaccination uptake. In some cases, people who did not receive vaccinations asserted that their bodies had the capability to fight off the virus without the use of medications. Based on this view, participants believe vaccination is less effective than natural immunity, thus reducing their desire to receive vaccinations [83].

Last but not least, despite the literature, risk perception plays a significant role in motivating health-centric behavior, especially in the case of pandemics. During COVID-19, we observed that mistrust of science partially mediated risk perception and vaccination intention (H3d). As Yaqub, Castle-Clarke [85] demonstrate, trust is one of the most important factors in determining whether a person is willing to receive vaccinations; credibility depends on one’s confidence in the source of information regarding a given health risk. When parents receive incorrect information about COVID-19, they become less concerned about the severity of the disease and are more likely to perceive a low risk of their children acquiring the disease. Therefore, parents may not see vaccination information as reliable simply because they do not trust the source of the information [86]. Jones, Omer [87] that parents with a low risk perception about COVID-19 might be more likely to seek information from other sources, such as the internet or other parents, if they mistrust science. Based on these findings, encouraging kids COVID-19 vaccinations may be futile if parents lack trust in science [85,88].

## 5. Limitations

A number of limitations are associated with this study. First and foremost, the use of an online sample may limit the generalizability of our results. We recommend that future research include a more diverse group of participants and revisit the relationships found in this study. In addition, since COVID-19 vaccinations were not widely available at the time of this study, the actual uptake of the vaccine was not assessed. Even though behavioral intentions and actual conduct tend to be closely correlated [89], we suggest testing the present paradigm with subsequent pandemic vaccinations and behavioral health interventions. Secondly, since we examined intentions rather than behavior, we cannot infer that parents’ desire to vaccinate their children translates into vaccination behavior. However, in light of the parents’ prior behavior towards vaccines (half of the parents refused to give their children mandatory vaccinations), there is a substantial correlation between intention and behavior. A number of other factors influence the intention to vaccinate; future research could include additional variables affecting religious morality, public health messages, misinformation, and mistrust of authorities.

## 6. Implications

As a result of the study, some practical implications can be drawn. In order to increase vaccination coverage during pandemics, epidemics, and outbreaks, policymakers must evaluate how self-reported health affects vaccine acceptability. The use of public health messages based on the perspectives of individuals, families, and communities may be necessary as complementary treatments to improve risk perception and vaccination intentions. In order to increase the effectiveness of vaccination messages, customized messages may be most effective. People may consider various reasons not to get vaccinated [90]. Despite the lack of specific information about vaccines, participants reported that news about side effects and rumors based on previous experiences prompted donors and policymakers to be cautious.

## 7. Conclusions

Our study examined the vaccination hesitancy of a representative sample of Pakistani parents, and three predominant reasons were identified. The first factor contributing to COVID-19 vaccine aversion is the belief that vaccines are subject to conspiracy theories, ignorance, and mistrust of science. Second, people are highly concerned about vaccine safety concerns since vaccines have been developed very rapidly. As a result, vaccine side effects are being viewed with a great deal of concern. The results of our research also indicated that participants were concerned about the spread of diseases and believed in the effectiveness of vaccinations in terms of disease reduction. According to the study, low perceived self-efficacy, lack of geographical accessibility, and low literacy contributed to low uptake, even when mobile campaigns and free services were provided. Third, to enhance convenience, participants suggested that vaccinations should be available at more convenient times and locations. To increase acceptance, educational and vaccination efforts should be coordinated with community preferences, such as weekend deliveries, door-to-door deliveries, and distribution by local volunteers. In order to reduce the spread of misinformation and mistrust, we believe that education must provide information regarding the purpose of vaccinations and potential side effects. The formation of community advisory boards consisting of citizens from the same district would also increase openness and information dissemination to communities.

## Figures and Tables

**Table 1:** Descriptive statistics.

Characteristics	Frequency	Percentage (%)
**Gender**		
Male	310	68.3
Female	144	31.7
**Parent’s Education**		
High School	38	8.4
College and Diploma	14	3.1
Graduation	117	25.8
Masters	249	54.8
**No. of Kids**		
1–2	294	64.8
3–5	119	26.2
6–8	32	7
More then 8	9	2
**Kid’s Age**		
Less than one year	105	23.1
1–3 year	144	31.7
4–6 year	90	19.8
7–9 year	55	12.1
**Parent’s Income (Annual)**		
$1000–10,000	380	83.7
$11,000–15,000	13	7.7
$16,000–20,000	35	5.7
Above $20,000	26	2.9
**Parent’s Profession**		
Educationist	99	21.8
HCWs	36	7.9
Engineers	154	33.9
Govt. Employee	52	11.4
Manager	54	11.9
Others	38	8.4
**Urbanicity**		
Rural	128	28.2
Urban	326	71.8
**Religion**		
Islam	441	97.1
Christian	7	1.5
Others	6	1.3
**Past Vaccine Experience**		
Yes	291	64.2
No	163	35.8

**Table 2:** Factor loadings and Cornbach Alpha.

Construct	Items	Loadings	CA
Parental Vaccine Intention (PVI)	PIV1	0.73	0.845
	PIV2	0.692	
	PIV3	0.643	
	PIV4	0.744	
Conspiracy Belief (CB)	CB2	0.725	0.908
	CB3	0.739	
	CB4	0.909	
	CB6	0.886	
	CB7	0.73	
	CB9	0.79	
	CB10	0.874	
	CB11	0.779	
Risk Perception(RP)	RP1	0.715	0.958
	RP2	0.744	
	RP3	0.846	
	RP4	0.894	
	RP5	0.93	
	RP6	0.962	
	RP7	0.985	
Vaccine Knowledge(VK)	VK2	0.752	0.902
	VK3	0.889	
	VK4	0.925	
	VK5	0.894	
	VK6	0.679	
Vaccine Safety Concerns(VS)	VS1	0.898	0.875
	VS2	0.914	
	VS3	0.748	
	VS4	0.664	
Mistrust in Science(MTS)	MTS1	0.539	0.710
	MTS2	0.673	
	MTS3	0.797	
	MTS4	0.847	
	MTS5	0.597	
Perceived Vaccine Self Efficacy(PVSE)	PVSE1	0.865	0.745
	PVSE2	0.968	
	PVSE3	0.904	
	PVSE4	0.623	

**Table 3:** Reliability, Validity and Discriminant validity.

.	CR	AVE	CB	VK	VS	RP	PIV	PVSE	MTS
**CB**	0.942	0.700	**0.837**						
**VK**	0.904	0.653	−0.067	**0.808**					
**VS**	0.880	0.652	−0.054	0.552 ***	**0.807**				
**RP**	0.943	0.808	−0.729 ***	0.025	0.010	**0.899**			
**PIV**	0.860	0.615	−0.125 *	0.364 ***	−0.218 ***	−0.170 ***	**0.784**		
**PVSE**	0.753	0.680	−0.030	−0.154 **	−0.191 ***	−0.076	0.131 *	**0.728**	
**MTS**	0.724	0.64	0.089†	−0.270 ***	−0.153 **	−0.046	0.193 ***	0.090	**0.696**

Composite Reliability (CR), Average extracted Varaince (AVE). Conspiracy belief (CB), Vaccine Knowledge (VK), Vaccine Safety (VS), Risk Perception (RP), Parental Vaccine Intention (PIV), Parental Vaccine Self-Efficacy (PVSE) and Mistrust in Science (MTS). *** *p* < 0.001. ** *p* < 0.010. * *p* < 0.050.

**Table 4:** Correlation Matrix among the Variables.

	RP	CB	MTS	VK	VS	PVSE	PIV
**RP**	1						
**CB**	0.654 **	1					
**MTS**	0.283 ***	0.382 *	1				
**VK**	−0.365 *	-0.25 **	−0.208 **	1			
**VS**	0.329 *	0.42 **	0.105 *	−0.459 **	1		
**PVSE**	−0.488 *	-0.426 **	−0.288	0.437 **	0.354 **	1	
**PIV**	−0.451 **	-0.495 ***	−0.324 **	0.520 **	−0.218 **	0.487 **	1

*** Correlation is significant at the 0.001 level (2-tailed). ** Correlation is significant at the 0.01 level (2-tailed). * Correlation is significant at the 0.05 level (2-tailed). * Conspiracy belief (CB), Vaccine Knowledge (VK), Vaccine Safety (VS), Risk Perception (RP), Parental Vaccine Intention (PIV), Parental Vaccine Self-Efficacy (PVSE) and Mistrust in Science (MTS).

**Table 5:** Standardized coefficient estimates for paths predicting parents’ intention towards their kid’s COVID-19 vaccination.

Direct Effect	Beta	SE	CR	*p*-Value
CB→PIV	−0.452	0.042	2.967	0.01 **
PVSE→PIV	0.368	0.041	1.969	0.090
VS→PIV	−0.233	0.046	2.889	0.075
RP→PIV	0.241	0.056	4.135	0.005 *
VK→PIV	0.522	0.055	14.343	***
MTS→PIV	−0.151	0.056	4.135	***

Significance of Estimates. *** *p* < 0.001. ** *p* < 0.010. * *p* < 0.050. * Conspiracy belief (CB), Vaccine Knowledge (VK), Vaccine Safety (VS), Risk Perception (RP), Parental Vaccine Intention (PIV), Parental Vaccine Self-Efficacy (PVSE) and Mistrust in Science (MTS) about COVID-19.

**Table 6:** Total, direct and indirect effects of psychosocial factors, Vaccine Knowledge, Mistrust in science on parents’ Intention towards their kid’s COVID-19 vaccination.

	Total Effect		Direct Effect			Indirect Effects			
	Beta	*p*-Value	Beta(*β)*	*p*-Value		Beta *(β)*	*p*-Value	CI 95%	Decision
CB→PIV	−0.475	0.046 *	−0.192	0.073	CB →VK→PIV	−0.283	0.002 **	[−0.127,−0.06]	Full
PVSE→PIV	0.261	0.002 **	0.102	0.006 **	PVSE→VK→PIV	0.159	0.015 *	[0.099,0.234]	partial
VS→PIV	−0.226	0.002 **	−0.206	0.001 ***	VS→VK→PIV	−0.020	0.619	[−0.063,0.019]	No medition
RP→PIV	0.137	0.014 **	0.097	0.100	RP→VK→PIV	0.088	0.002 **	[0.042,0.139]	Full
CB→PIV	0.215	0.038 *	0.074	0.059	CB→MTS→PIV	0.1419	0.031 *	[0.02,0.263]	Full
PVSE→PIV	0.275	0.002 **	0.173	0.002 **	PVSE→MTS→PIV	0.102	0.110	[−0.245,0.356]	No medition
VS→PIV	−0.205	0.001 ***	−0.195	0.002 **	VS→MTS→PIV	−0.010	0.047 *	[−0.341,−0.031]	Partial
RP→PIV	−0.149	0.011 **	−0.138	0.014 **	RP→MTS→PIV	−0.0112	0.042 *	[−0.36,−0.062]	Partial

**Note**: Bootstrapped 95% confidence interval (N = 2000), Significance of Estimates. *** *p* < 0.001. ** *p* < 0.010. * *p* < 0.050. * Conspiracy belief (CB), Vaccine Knowledge (VK), Vaccine Safety (VS), Risk Perception (RP), Parental Vaccine Intention (PIV), Parental Vaccine Self-Efficacy (PVSE) and Mistrust in Science (MTS).

## Data Availability

The data that support the findings of this study are available from the corresponding author, Riffat Shahani, upon reasonable request.
